# Darolutamide in Combination with Radium-223 Exhibits Synergistic Antitumor Efficacy in LNCaP Prostate Cancer Models

**DOI:** 10.3390/ijms252413672

**Published:** 2024-12-21

**Authors:** Urs B. Hagemann, Christoph A. Schatz, Mari I. Suominen, Andreas Schlicker, Matias Knuuttila, Timothy Wilson, Esa Alhoniemi, Sanna-Maria Käkönen, Bernard Haendler, Arne Scholz

**Affiliations:** 1 Bayer AG, Research & Development, Pharmaceuticals, 13353 Berlin, Germany; 2 Pharmatest Services Ltd., 20520 Turku, Finland; 3 Aurexel Life Sciences Ltd., 21240 Askainen, Finland; 4 Inoi Oy, 20100 Turku, Finland; 5 Institute of Biomedicine, University of Turku, 20520 Turku, Finland

**Keywords:** radium-223, darolutamide, prostate cancer, bone metastases, CRPC

## Abstract

Despite treatment, prostate cancer commonly progresses into castration-resistant prostate cancer (CRPC), which remains largely incurable, requiring the development of new interventions. Darolutamide is an orally administered second-generation androgen receptor inhibitor indicated for patients with non-metastatic CRPC or metastatic hormone-sensitive prostate cancer. Here, we evaluated the effect of androgen receptor (AR) inhibition by darolutamide in combination with DNA double-strand-break-inducing targeted radium-223 alpha therapy in vitro and in an intratibial LNCaP xenograft model mimicking prostate cancer metastasized to bone. The results highlight the synergistic antitumor efficacy of darolutamide in combination with radium-223 both in vitro and in vivo. This effect was most likely driven by the downregulation of genes involved in DDR signaling, which was demonstrated in vitro by a gene set enrichment analysis. The combination treatment also reduced pathological tumor-induced effects in bone by decreasing the number of osteoblasts and osteoclasts and reducing abnormal bone formation in tumor-bearing bone. Additionally, it was shown that darolutamide does not affect the uptake of radium-223 into bone tissue. These results support the investigation of darolutamide in combination with radium-223 for the treatment of patients with CRPC metastasized to bone.

## 1. Introduction

Prostate cancer is the second most frequently diagnosed cancer in men in developed countries [1]. Most patients will initially respond to therapy, but eventually, the disease becomes resistant to therapy and progresses into castration-resistant prostate cancer (CRPC) [2,3]. Despite recent progress in the treatment of prostate cancer, metastatic CRPC (mCRPC) remains a largely incurable disease, and novel efficient treatment options are required.

Darolutamide is an orally administered second-generation androgen receptor inhibitor indicated for patients with metastatic hormone-sensitive prostate cancer (mHSPC) [4,5] or non-metastatic castration-resistant prostate cancer (nmCRPC) [6]. Previous studies have demonstrated that AR inhibition with agents such as darolutamide results in the downregulation of DNA damage repair (DDR) genes in prostate cancer [7,8,9]. The efficacy of darolutamide in the treatment of prostate cancer, combined with its ability to downregulate DDR genes, makes it a viable candidate for use in a combination treatment regimen together with a DNA damage-inducing therapeutic agent.

Radium-223 dichloride (radium-223, Xofigo^®^) is a targeted alpha therapy (TAT) that improves overall survival and quality of life and reduces symptomatic skeletal events (SSEs) in patients with CRPC metastasized to bone [10,11]. It is the first and currently only targeted alpha therapy available for metastatic CRPC (mCRPC) and targets bone metastases associated with increased bone turnover by incorporating into newly formed bone and inducing DNA double-strand breaks (DSBs) in cancer cells, as well as in osteoblasts and osteoclasts, via alpha radiation [12,13].

In this study, we evaluated the potential synergistic effects of AR inhibition and targeted alpha therapy by studying the antitumor effects of darolutamide in combination with radium-223 in LNCaP prostate cancer cells in vitro, as well as in an intratibial LNCaP xenograft model mimicking prostate cancer metastasized to bone. Although LNCaP cells are considered androgen-sensitive in vitro, they are derived from a patient with metastatic CRPC and harbor AR mutation that confers resistance to castration [14]. This article is a revised and expanded version of a conference paper entitled “Radium-223 in combination with darolutamide exhibits synergistic antitumor efficacy in LNCaP prostate cancer models”, which was presented at the AACR Annual Meeting, 5–10 April 2024, San Diego, CA, USA [15]. The results support the ongoing clinical development of darolutamide in combination with radium-223-based TAT for the treatment of mCRPC.

## 2. Results

### 2.1. Darolutamide Sensitizes LNCaP Cells to Radium-223 Treatment In Vitro

**Figure 1:**
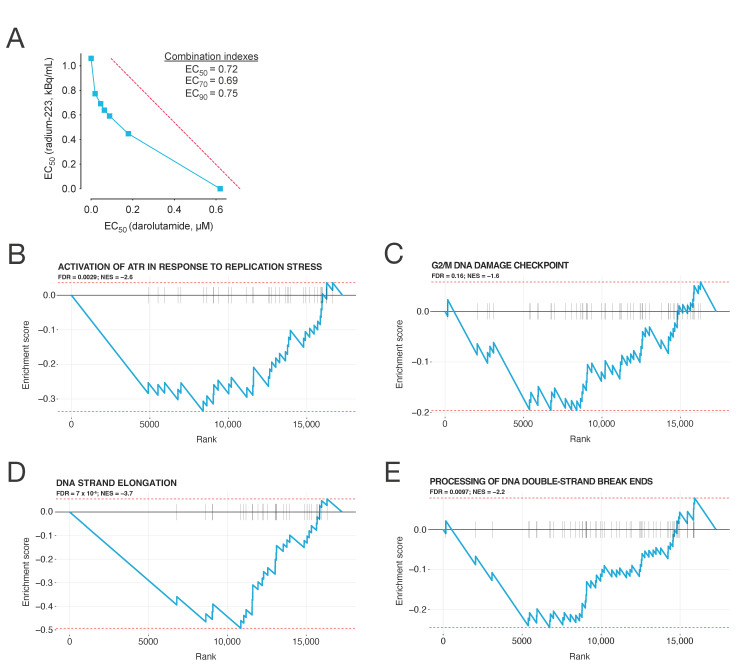
Darolutamide sensitizes LNCaP cells to radium-223 treatment in vitro. (**A**) Isobologram showing the combination effects of darolutamide and radium-223 on the cell viability of LNCaP prostate cancer cells. Combination indexes were calculated for the combination treatment of darolutamide and radium-223, with 0.70–0.85 defined as moderate synergism and 0.3–0.7 defined as synergism. Enrichment plots for the Reactome pathways: (**B**) activation of ATR in response to replication stress, (**C**) G2/M DNA damage checkpoint, (**D**) DNA strand elongation, and (**E**) processing of DNA double-strand break ends. Effect size and statistical significance are indicated by the normalized enrichment score (NES) and false discovery rate (FDR).

The viability of androgen-sensitive LNCaP cells was evaluated by exposing them to darolutamide and radium-223 under androgen-depleted conditions in vitro. The combination of darolutamide and radium-223 showed a suppressive effect on cell proliferation with moderate synergism, with combination indexes between 0.69 and 0.75 (Figure 1A). The single-compound EC_50_ values for darolutamide and radium-223 were 7.72 µM and 1.03 kBq/mL, respectively.

To evaluate the impact of darolutamide on the genes involved in repairing DNA damage, DDR pathways were studied in LNCaP cells treated with the synthetic androgen R1881 alone or in combination with darolutamide. A gene set enrichment analysis (GSEA) revealed a prominent downregulation of pathways involved in DNA damage response (Figure 1B–E).

### 2.2. Darolutamide Potentiates the Antitumor Efficacy of Radium-223 In Vivo

**Figure 2:**
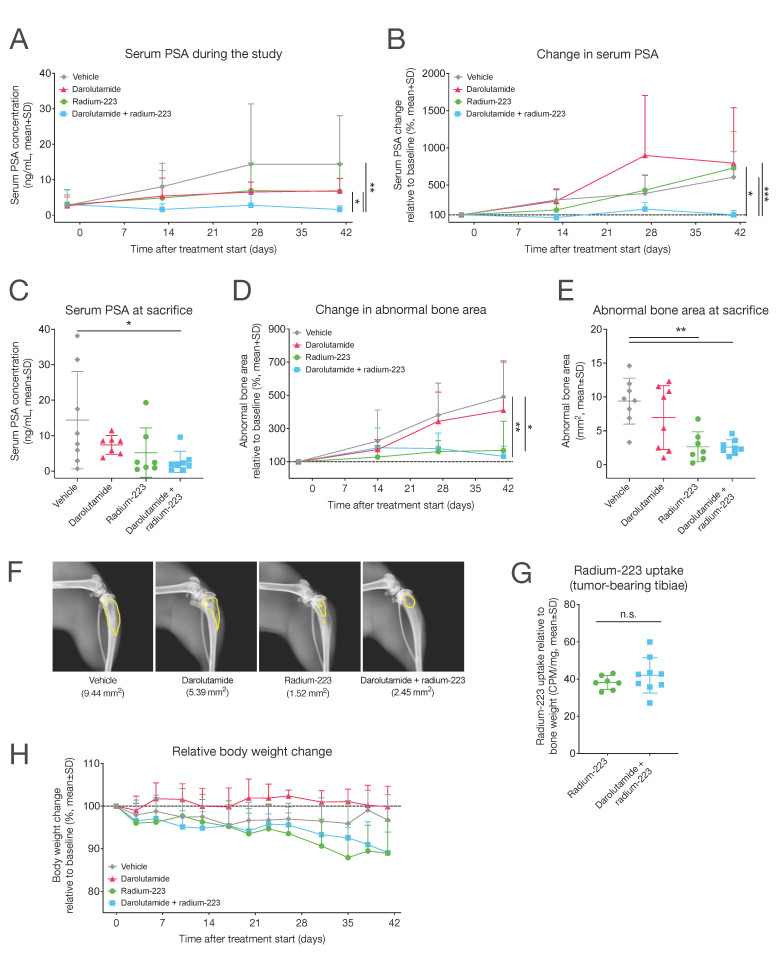
Darolutamide potentiates the antitumor efficacy of radium-223 in vivo. Blood samples were collected from the saphenous vein of mice (*n* = 7–9/group) 2 days prior to and 13, 27, and 41 days after the initiation of treatment. The mice were treated with vehicle; darolutamide (100 mg/kg, BID, p.o.); radium-223 (330 kBq/kg, Q4Wx2, i.v.); or a combination of darolutamide and radium-223 and were sacrificed on day 41 after the start of treatment, except for one mouse in the radium-223 monotherapy group and two mice in the radium-223 and darolutamide combination group, which were sacrificed on day 38. (**A**) Absolute and (**B**) relative serum PSA change during the study and (**C**) absolute PSA concentrations at sacrifice. Total area of abnormal bone measured by radiography, presented as (**D**) relative abnormal bone area change during the study and (**E**) absolute values at sacrifice. (**F**) Representative ex vivo X-ray images of tumor-bearing tibiae. Areas of abnormal bone growth are marked with yellow outlines, and the values indicate the area of abnormal bone measured within these outlines. (**G**) Radium-223 uptake in bone, determined by measuring radioactivity in tumor-bearing tibiae. The results are expressed as counts per minute (CPM) normalized to the bone sample weight. (**H**) Body weights of mice recorded two times a week during the treatment period and presented relative to the pre-treatment baseline. Plots describe mean and standard deviation (SD). Statistical analyses were performed using mixed models and model contrasts (**A**,**B**,**D**,**H**), ANOVA followed by contrasts (**C**,**E**), or Welch’s *t*-test (**G**): *, *p <* 0.05; **, *p <* 0.01; ***, *p <* 0.001; n.s., non-significant.

The antitumor efficacy of darolutamide in combination with radium-223 was studied using an intratibial LNCaP xenograft mouse model mimicking the growth of bone metastatic prostate cancer. The mice were treated with vehicle; darolutamide (100 mg/kg, BID, p.o.); radium-223 (330 kBq/kg, Q4Wx2, on days 0 and 28, i.v.); or their combination for 41 days. At the time of randomization, the mean PSA value of mice allocated to the treatment groups was 2.88 ng/mL (range: 0.17–14.2 ng/mL). Darolutamide in combination with radium-223 exhibited a synergistic antitumor efficacy in vivo, as observed by the lower PSA concentrations when compared with vehicle (*p* = 0.005), darolutamide (*p* = 0.002), or radium-223 (*p* = 0.012) monotherapies (Figure 2A). Increasing PSA concentrations in serum were observed in mice treated with vehicle, radium-223, or darolutamide monotherapies, but PSA concentrations in the monotherapy groups remained below the vehicle levels (Figure 2A). In contrast, mice treated with darolutamide in combination with radium-223 prevented the PSA increase throughout the study, as the relative mean PSA change at sacrifice was 102% of the pre-treatment level, indicating inhibitory tumor control by the combination treatment (Figure 2B). This relative mean PSA change was only 16.7% of the corresponding PSA change in the vehicle group. Furthermore, a statistical interaction between the darolutamide and radium-223 treatments was found (*p* = 0.04), confirming the observed synergistic effect.

At sacrifice, mice treated with the combination treatment of darolutamide and radium-223 had lower PSA values (*p* = 0.03) compared with mice treated with the vehicle (Figure 2C). The radium-223 monotherapy and the combination treatment of darolutamide and radium-223 were efficacious in inhibiting tumor-induced abnormal bone growths in tumor-bearing tibiae when compared with vehicle, both throughout the treatment period (Figure 2D) and at sacrifice (Figure 2E,F). Concurrent treatment with darolutamide did not alter the uptake of radium-223 in tumor-bearing mice (Figure 2G). Although a slight decline in relative body weight change was observable at the end of the treatment period in the radium-223 monotherapy and combination treatment groups (Figure 2H), no drastic changes in body weight were observed during the treatment period, demonstrating that the treatments were well tolerated.

### 2.3. Darolutamide in Combination with Radium-223 Inhibits Abnormal Bone Turnover

**Figure 3:**
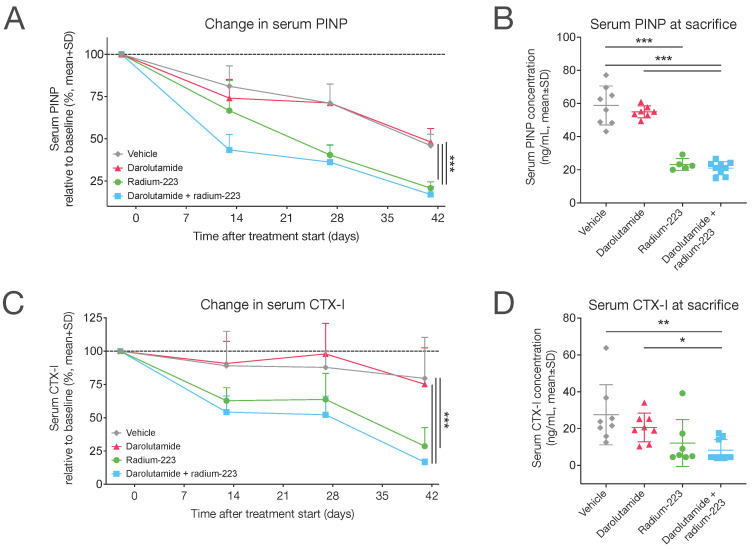
Darolutamide in combination with radium-223 inhibits abnormal bone turnover. The levels of the bone formation marker (**A**,**B**) PINP and bone resorption marker (**C**,**D**) CTX-I were measured in blood samples collected from the saphenous vein of mice (*n* = 7–9/group) 2 days prior to and 13, 27, and 41 days after the initiation of treatment. The mice were treated with vehicle; darolutamide (100 mg/kg, BID, p.o.); radium-223 (330 kBq/kg, Q4Wx2, i.v.); or a combination of darolutamide and radium-223 and were sacrificed on day 41 after the start of treatment, except for one mouse in the radium-223 monotherapy group, which was sacrificed on day 38. Plots describe mean and standard deviation (SD). Statistical analyses were performed using mixed models and model contrasts (**A**,**C**), ANOVA followed by contrasts (**B**), or a Kruskal–Wallis test followed by Dunn’s pairwise comparison test (**D**): *, *p* < 0.05; **, *p* < 0.01; ***, *p* < 0.001.

To assess the impact of darolutamide and radium-223 on bone turnover, we examined the levels of two bone turnover markers in serum: procollagen type I N-terminal propeptide (PINP) and C-terminal telopeptide of type I collagen (CTX-I). These markers are associated with bone formation and bone resorption, respectively. Notably, mice treated with radium-223 monotherapy (28.1% of the pre-treatment level) or a combination of darolutamide and radium-223 (17.0% of the pre-treatment level) displayed a marked reduction in PINP levels compared with vehicle (*p* < 0.001) (Figure 3A). However, all groups showed a decreasing trend in PINP levels (<50% of the pre-treatment level) over the course of the study (Figure 3A). Mice receiving the combination treatment exhibited remarkably lower PINP levels at the end of the study compared with those treated with vehicle (*p* < 0.001) or darolutamide monotherapy (*p* < 0.001) (Figure 3B). As observed over the course of treatment, CTX-I levels in serum were also decreased in mice treated with the combination treatment of darolutamide and radium-223 (Figure 3C), resulting in lower CTX-I levels at sacrifice (Figure 3D) when compared with mice treated with vehicle (*p* = 0.005) or darolutamide monotherapy (*p* = 0.025). All in all, these results indicate that the concurrent use of darolutamide does not interfere with the potential of radium-223 to hinder abnormal bone metabolic activity.

### 2.4. Darolutamide Enhances the Inhibiting Effects of Radium-223 on Tumor-Induced Bone Formation

**Figure 4:**
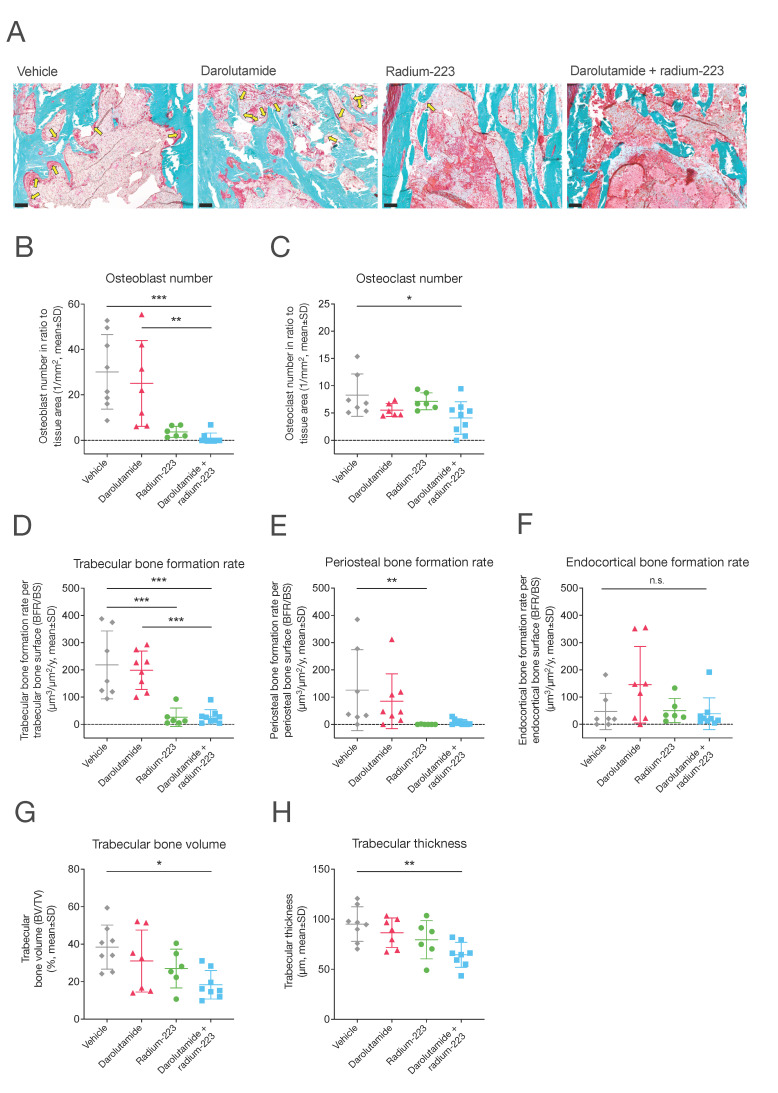
Darolutamide enhances the inhibiting effects of radium-223 on tumor-induced bone formation. The mice (*n* = 6–9/group) were treated with vehicle; darolutamide (100 mg/kg, BID, p.o.); radium-223 (330 kBq/kg, Q4Wx2, i.v.); or a combination of darolutamide and radium-223 and were sacrificed on day 41 (after the start of treatment), except for one mouse in the radium-223 monotherapy group and two mice in the radium-223 and darolutamide combination group, which were sacrificed on day 38. The bones were labeled with calcein green and alizarin red in vivo to measure the dynamic histomorphometry parameters. (**A**) Representative images (10×) of osteoblast clusters (indicated with yellow arrows) in tumor-bearing tibiae in all treatment groups, visualized by Masson–Goldner trichrome staining. Scale bar length: 100 μm. The number of (**B**) osteoblasts and (**C**) osteoclasts on the trabecular bone surface relative to the tissue area in tumor-bearing tibiae. Bone formation rate is described as (**D**) trabecular, (**E**) periosteal, and (**F**) endocortical bone formation rate per trabecular, periosteal, and endocortical bone surface (BFR/BS) in tumor-bearing tibiae, respectively. (**G**) Trabecular bone volume (percent bone volume, BV/TV) and (**H**) trabecular thickness in tumor-bearing tibiae. Plots describe mean and standard deviation (SD). Statistical analyses were performed using ANOVA followed by contrasts (**B**–**D**,**G**,**H**) or a Kruskal–Wallis test followed by Dunn’s pairwise comparison test (**E**,**F**): *, *p <* 0.05; **, *p <* 0.01; ***, *p <* 0.001; n.s., non-significant.

Next, we studied whether darolutamide in combination with radium-223 affects bone formation, reflecting tumor-induced changes in bone. Using bone histomorphometry, the bone formation, bone remodeling, and cellular characteristics in tumor-bearing tibiae were quantitatively evaluated. The number of disease-driving osteoblasts relative to the tissue area was drastically reduced by both the radium-223 monotherapy and the combination treatment of darolutamide and radium-223 compared to vehicle (Figure 4A,B). A minor decrease in the osteoclast number relative to the tissue area was also observed in the combination group when compared with vehicle (Figure 4C). As one might expect, radium-223 decreased the trabecular bone formation rate in tumor-bearing tibiae both in the absence and presence of darolutamide (Figure 4D). A similar effect was observed on the periosteal bone formation rate with the radium-223 monotherapy but not in combination with darolutamide (Figure 4E). It is worth noting that none of the treatments affected the endocortical bone formation rate (Figure 4F). Mineral apposition rates and mineralizing surfaces in trabecular, periosteal, and endocortical bone were also assessed (Figure S1A–F), and the results are comparable to the respective bone formation rates. Interestingly, both the trabecular bone volume (*p* = 0.011) (Figure 4G) and trabecular thickness (*p* = 0.003) (Figure 4H) were also decreased by the combination treatment of darolutamide and radium-223, while the radium-223 or darolutamide monotherapies did not demonstrate such effects.

## 3. Discussion

Darolutamide has been used for the treatment of mHSPC in combination with androgen-deprivation therapy with or without the addition of docetaxel, and data obtained from a phase 3 clinical trial demonstrate that this therapy regimen significantly improves survival in mHSPC patients [16,17]. While this treatment delays the progression of the disease, the onset of mCRPC is generally inevitable, as tumor cells adapt to low androgen levels [2,3]. At this point, androgen receptor inhibitors and targeted alpha therapy are common treatment options, as they both have been shown to improve overall survival and quality of life and to reduce symptomatic skeletal events in patients with mCRPC [10,18]. While these therapies are usually used as single agents, there is compelling evidence that combination treatment could provide synergistic benefits in the treatment of mCRPC [9,19]. Interestingly, the practice changing results from PEACE III, a clinical trial combining radium-223 and enzalutamide, were recently published, demonstrating that adding six cycles of radium-223 significantly improved radiographic progression-free survival in patients with mCRPC treated with enzalutamide as a first-line therapy [20]. In addition to this, an interim analysis showed a statistically significant overall survival benefit, favoring enzalutamide in combination with radium-223 [20]. This interim analysis includes 80% of events and is to be confirmed via a final overall survival analysis.

In this study, we demonstrated the synergistic antitumor efficacy of darolutamide in combination with radium-223 both in vitro and in vivo. It is hypothesized that this effect results from sensitization to radiation therapy caused by darolutamide-induced AR inhibition. Recent research has shown that radiation therapy in combination with the AR inhibitor enzalutamide decreases the survival of AR-positive LNCaP cells but not of AR-negative PC-3 prostate cancer cells [21]. Additionally, both androgen deprivation therapy and AR inhibitors can suppress DNA damage repair genes, and 32 genes associated with DNA damage repair have been identified to be androgen-regulated in LNCaP cells [8]. Furthermore, a number of in vitro and in vivo prostate cancer models, including LNCaP cell lines expressing wild-type or mutated AR, have shown that radiation therapy upregulates AR signaling in CRPC, leading to radioresistance [22]. Thus, it is probable that a concurrent treatment with darolutamide makes cancer cells more vulnerable to radium-223-induced DNA DSBs.

To support the hypothesis that radiosensitization lies behind the synergistic antiproliferative effects of darolutamide in combination with radium-223, we explored DDR signaling-related pathways in LNCaP cells treated with the synthetic androgen R1881 alone or in combination with darolutamide. Using RNA sequencing, the GSEA demonstrated a prominent darolutamide-induced downregulation in DDR signaling-related pathways. A nearly identical effect has also recently been demonstrated in LNCaP cells in a similar test setup using radium-223 and enzalutamide treatments [9]. Additionally, previous studies combining PSMA-targeted alpha therapies, either thorium-227-HOPO-pelgifatamab (^227^Th-pelgi) or actium-225-macropa-pelgifatamab (^225^Ac-pelgi), and darolutamide have shown enhanced antitumor efficacy in preclinical prostate cancer models, which is consistent with the darolutamide-induced suppression of DDR signaling [7,23]. Taken together, these results suggest that the synergistic effects of darolutamide in combination with radium-223 arise from the radiosensitization of cancer cells by the darolutamide-induced downregulation of DDR pathways.

In the intratibial LNCaP xenograft model, the effects of the combination of darolutamide and radium-223 were prominent, along with decreases in both serum PSA levels and areas of tumor-induced abnormal bone, while radium-223 or darolutamide alone did not reduce serum PSA levels. We previously demonstrated that radium-223 at a similar dose as in this study had limited antitumor efficacy on PSA in the intratibial LNCaP model [24]. However, here, only a statistically non-significant decreasing trend was observed, highlighting the synergistic effect of darolutamide on radium-223 treatment. In another previous preclinical study with the intratibial LNCaP model, radium-223 in combination with abiraterone and prednisone did not exhibit additive/synergistic antitumor effects [25]. The lack of efficacy might be caused by an abiraterone/prednisone-mediated reduction in radium-223 uptake to abnormal bone. However, in the present study, darolutamide was demonstrated to have no effect on radium-223 uptake.

Both darolutamide in combination with radium-223 as well as radium-223 treatment alone showed a significant impact on bone turnover, as indicated by decreased levels of the bone formation marker PINP and the bone resorption marker CTX-I. Decreased levels of bone turnover markers, including PINP, have been demonstrated to be associated with improved outcomes in mCRPC patients in a phase 2 trial [26]. This result is also in line with previous research, which has shown that radium-223 decreases PINP levels in serum, reflecting the inhibition of pathological bone changes in the LNCaP model [24]. As a monotherapy, darolutamide did not have any effect on PINP or CTX-I levels. It should be noted, however, that both treatment-induced and cancer-induced changes in bone turnover are reflected by these bone markers. Moreover, during treatment, the mice were at an age where their normal bone growth slows down, which can also affect bone marker levels as well as the interpretation of these results.

The reduction in tumor-induced abnormal bone growth was confirmed by dynamic bone histomorphometry, which revealed that darolutamide in combination with radium-223 decreased the trabecular bone formation rate. A similar effect on the periosteal bone formation rate was also observed with the radium-223 monotherapy; however, none of the treatments affected the endocortical bone formation rate. No major bone microarchitecture-compromising effects were observed, but it remains unknown whether the decreases in bone formation contribute to a risk of non-pathologic fractures. Nevertheless, recent studies do suggest that radium-223 treatment does not increase the risk of fractures to any significant extent when it is administered in combination with either enzalutamide [27,28], denosumab, or zoledronic acid [29]. In addition to the reduced bone formation, we also observed a decrease in the number of osteoblasts as well as a minor decrease in the number of osteoclasts in tumor-bearing tibiae following treatment with darolutamide in combination with radium-223. Interestingly, such a decrease in osteoclast number was not observed with enzalutamide in a previous study when the second dose of radium-223 was given just one day before sacrifice [9]. However, in this study, radium-223 was administered 13 days before sacrifice, and this different schedule is likely to explain this observation.

In conclusion, this study demonstrated the enhanced antitumor efficacy of darolutamide in combination with radium-223 in vitro and in an in vivo xenograft model mimicking prostate cancer metastasized to bone. The combination treatment also reduced pathological tumor-induced effects in bone by decreasing the number of osteoblasts and osteoclasts and reducing abnormal bone formation in tumor-bearing bone. Importantly, a concurrent administration of darolutamide did not affect the uptake of radium-223 into bone tissue, which possibly explains the synergistic effects of the combination treatment. These results support the investigation of darolutamide in combination with radium-223 for the treatment of patients with CRPC metastasized to bone.

## 4. Materials and Methods

### 4.1. Cell Viability Assay

The cell viability of androgen-sensitive LNCaP human prostate cancer cells was measured using a CellTiter-Glo^®^ assay (Promega, Madison, WI, USA) after 6 days of exposure to darolutamide and radium-223. Isobolograms and combination indexes were calculated as described by Chou-Talalay [30], with a combination index of <0.8 defined as a synergistic effect. EC_50_, EC_70_, and EC_90_ values were computed for every individual combination data point. Isobolograms were generated, and the results were subsequently confirmed in a follow-up experiment. Additional information regarding the assay protocol is described in the Supplementary Methods.

### 4.2. RNA Sequencing and Gene Set Enrichment Analysis

First, LNCaP cells were grown for 48 h in a medium supplemented with 10% charcoal-stripped fetal bovine serum (FBS). The cells were treated with the synthetic androgen R1881 and darolutamide at final concentrations of 1 µM and 2 µM, respectively, and collected 22 h after treatment. Subsequently, the cells were lysed, and RNA extraction was performed using RNeasy columns (Qiagen, Hilden, Germany) with on-column DNA digestion, following the manufacturer’s instructions. RNA integrity was assessed using an Agilent 2100 Bioanalyzer (Agilent, Santa Clara, CA, USA), and samples with RNA Integrity Number (RIN) values exceeding eight underwent further processing. Following mRNA purification with poly-T beads, RNA library preparation was carried out according to the manufacturer’s instructions (TruSeq Stranded mRNA Kit; Illumina, San Diego, CA, USA).

Biological replicates (5 for R1881 treatment and 10 for the combination of R1881 and darolutamide) were subsequently subjected to sequencing on a HiSeq 2500 instrument (Illumina) utilizing 50 single-end base-pair reads, with an average sequencing depth of 21 million reads per sample. FASTQ reads were aligned to the human genome GRCh38 using the STAR aligner and quantified using featureCounts from the Subread package [31]. Within each treatment group, the gene expression was summarized as the median count per million (CPM) values. The gene set enrichment analysis (GSEA) [32] focused on Reactome pathways [33] from the Molecular Signatures Database, version 2022.1 [34], including pathways with a gene count ranging from 15 to 500, and was conducted using the Fast Gene Set Enrichment (fgsea) package, version 1.22.0 [35], comparing the ratio of median CPM values between R1881 treatment and the combination treatment of R1881 and darolutamide. Pathways were considered differentially regulated if their adjusted p-values were less than 0.05. The sequencing data were uploaded to the Gene Expression Omnibus (GEO) database and are publicly available (accession number: GSE280187).

### 4.3. Intratibial LNCaP Xenograft Model

Androgen-sensitive LNCaP cells, which secrete prostate-specific antigen (PSA) [36], were inoculated (2 × 10^6^ cells in 20 µL PBS) into the bone marrow cavity of the right proximal tibia of 5–6-week-old male NOD.scid mice (NOD.CB17/Prkdcscid/scid/Rj, Janvier Laboratories, France), eventually forming mixed osteoblastic/osteolytic lesions typical of patients with bone metastatic prostate cancer [24]. Approximately six weeks after inoculation, mice were stratified to treatment groups using serum PSA and radiographic bone scoring (*n* = 7–9/group).

**Figure 5:**
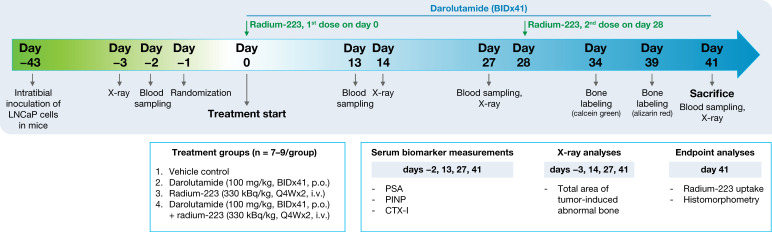
Timeline of the intratibial LNCaP model. Six weeks after inoculation, the mice were stratified to treatment groups and treated with vehicle; darolutamide (100 mg/kg, BIDx41, p.o.); radium-223 (330 kBq/kg, Q4Wx2, i.v., on days 0 and 28); or a combination of darolutamide and radium-223 for 41 days.

Mice were treated with either vehicle; darolutamide (100 mg/kg, twice daily (BID) with a 6-h interval, p.o.); radium-223 (330 kBq/kg, every four weeks with two doses (Q4Wx2), i.v.); or a combination of darolutamide (100 mg/kg, BID, p.o.) and radium-223 (330 kBq/kg, Q4Wx2, i.v.) for 41 days (Figure 5). The vehicles for darolutamide or radium-223 were solutions containing 50% PEG400, 30% propylene glycol, and 20% glucose (5% solution) at pH 7.5–8 or 28 mmol/L sodium citrate, respectively. Animal experiments were approved by the Animal Experiment Board in Finland (license number: ESAVI-8061/2020), and all experiments were conducted according to the guidelines of the European Union directive 2010/63/EU. Additional procedures are described in the Supplementary Methods.

### 4.4. Serum PSA and Bone Turnover Markers

Blood samples (100–200 μL) were collected from the saphenous vein after six hours of fasting one day before randomization and every other week after the initiation of treatments. At sacrifice, blood samples were obtained through a cardiac puncture. The samples were collected into Microvette 200 Z-Gel tubes (Sarstedt Ag & Co., Nümbrecht, Germany) and were gently inverted. The blood was allowed to clot at room temperature for 30–60 min, followed by centrifugation at 10,000× *g* at room temperature for 5 min. The serum samples were stored at −80 °C. Levels of serum PSA, the bone formation marker PINP, and the bone resorption marker CTX-I were measured at four time points: two days before treatment initiation and on days 13, 27, and 41 after treatment initiation. The markers were analyzed using a Human Kallikrein 3/PSA Quantikine^®^ enzyme-linked immunosorbent assay (ELISA) kit (R&D Systems, Minneapolis, MN, USA) and Rat/Mouse PINP and RatLaps™ CTX-I ELISA kits (both from IDS Ltd., Boldon, UK). These kits utilize quantitative sandwich or competitive ELISA techniques with peroxidase-linked antibodies specific to human PSA, PINP, or CTX-I and chromogenic (tetramethylbenzidine) color detection. Measurements were performed according to the manufacturers’ instructions. Serum samples for PSA, PINP, and CTX-I measurements were added to pre-coated microplates. Equivalent volumes of assay standards and controls were used. For quantification, absorbance was measured at 450 nm using a VICTOR2 Multilabel Counter (PerkinElmer, Waltham, MA, USA).

### 4.5. Radiography of Tumor-Bearing Tibiae

Tumor-bearing tibiae were imaged using a Faxitron Specimen Radiographic System (MX-20 D12) (Faxitron Corp., Wheeling, IL, USA) and the Faxitron Dicom software, version 3.0. The tumor-induced abnormal bone area was determined from the radiography (X-ray) images using the MetaMorph image analysis software, version 7.8.0.0 (Molecular Devices LLC, Sunnyvale, CA, USA).

### 4.6. Ex Vivo Analyses

At sacrifice, hind limbs were collected, and bone formation and cellular characteristics were analyzed by bone histomorphometry using an OsteoMeasure7 histomorphometry system (OsteoMetrics, Atlanta, GA, USA). The bone histomorphometry parameters are listed in Table S1. To evaluate radium-223 uptake in bone, the radioactivity of tumor-bearing and non-tumor-bearing tibiae was measured using an automatic gamma counter (Hidex, Turku, Finland). Tumor-bearing tibia histology sections were stained with H&E and analyzed using a Pannoramic 1000 slide scanner (3DHISTECH Ltd., Budapest, Hungary). The methods are described in detail in the Supplementary Methods.

### 4.7. Statistical Analyses

Statistical analyses were performed using the R statistical software, version 4.2.2 [37]. Longitudinal PSA, PINP, and CTX-I data were log-transformed and analyzed using mixed models and model contrasts. For PSA data, the values at the end of the study were analyzed relative to the baseline using ANOVA, and pairwise comparisons were conducted using ANOVA contrasts. The endpoint data for PINP and CTX-I and the histomorphometry data were analyzed using ANOVA followed by contrasts or a Kruskal–Wallis test followed by Dunn’s test. For the ex vivo radiography analysis, the data were analyzed using ANOVA after square root transformation. A Kruskal–Wallis test and pairwise comparisons using Dunn’s test were applied for histology analyses. The radium-223 uptake data were analyzed using Welch’s *t*-test. For each parameter, outliers were identified based on a simple robust estimation of the mean (using median) and standard deviation (using the scaled median absolute deviation) [38]. Values further than three standard deviations away from the mean were considered as outliers and removed from the data. All statistical tests used were two-sided. The obtained p-values were adjusted for all analyses.

## Data Availability

The data presented in this study are openly available in the Gene Expression Omnibus database (accession number: GSE280187) and in the article and in its online Supplementary Materials.

## Supplementary Materials

The supporting information can be downloaded at: https://www.mdpi.com/article/10.3390/ijms252413672/s1, References [39,40,41] are cited in the Supplementary Materials.
