# Supplementary material for: Darolutamide in Combination with Radium-223 Exhibits Synergistic Antitumor Efficacy in LNCaP Prostate Cancer Models
Source: Int J Mol Sci. 2024 Dec 21;25(24):13672. doi: 10.3390/ijms252413672 (PMC11677307; doi:10.3390/ijms252413672)
Supplement: Supplementary file 1 [file ijms-25-13672-s001.zip › ijms-3340885-supplementary.pdf]

SUPPLEMENTARY MATERIALS

Darolutamide in combination with radium-223 exhibits synergistic antitumor efficacy in LNCaP prostate cancer models

Urs B. Hagemann [1], Christoph A. Schatz [1], Mari I. Suominen [2], Andreas Schlicker [1], Matias Knuuttila [3], Timothy Wilson [3], Esa Alhoniemi [4], Sanna-Maria Käkönen [3,5], Bernard Haendler [1], Arne Scholz [1]

[1]Bayer AG, Research & Development, Pharmaceuticals, Berlin, Germany

[2]Pharmatest Services Ltd., Turku, Finland

[3]Aurexel Life Sciences Ltd., Askainen, Finland

[4]Inoi Oy, Turku, Finland

[5]Institute of Biomedicine, University of Turku, Turku, Finland

SUPPLEMENTARY METHODS

In vitro cell viability assay

LNCaP cells were cultured in standard cell culture conditions (+37°C, 5% CO_2) and seeded with a density of 1000 cells/well onto CulturPlate-384 well plates (#6007689, PerkinElmer, Waltham, MA, USA) in assay medium consisting of RPMI-1640 phenol-red free medium (FG 1215, Biochrom, Cambridge, UK) and 10% charcoal-stripped fetal bovine serum (cFBS). Next day, after adding fresh assay medium containing 0.3 nM R1881, darolutamide (0.04-20 µM) or radium-223 (0.04-20kBq/mL) were added to the 384-well plates using the D300e digital dispenser (Tecan, Männedorf, Switzerland). The cells were incubated for 6 days before the cell viability was determined using CellTiter-Glo® (CTG) assay (#G7573, Promega, Madison, WI, USA) by adding 30 µL of CTG reagent directly to the cells. The plates were put on a shaker (400 rpm) for 2 minutes and incubated for 8 minutes at RT. Luminescence was measured using a PHERAstar® FSX plate reader (BMG LABTECH, Ortenberg, Germany).

Compounds

For *in vivo* studies, darolutamide (Orion Corporation, Espoo, Finland) was dissolved in 50% PEG400, 30% propyleneglycol and 20% glucose (5% solution) pH 7.5-8, to reach a final concentration of 20 mg/mL. Radium-223 dichloride (radium-223) was synthesized at Bayer AG. For *in vivo* studies, radium-223 diluted with 28 mmol/L sodium citrate to form a solution with a radium-223 concentration of 66 kBq/mL

Cell culture for *in vivo* studies

LNCaP cells human prostate cancer cells (CRL-1740™, ATCC) were cultured in standard cell culture conditions and authenticated using short tandem repeat analysis (GenePrint10 system, Promega, Madison, WI, USA) at the Institute for Molecular Medicine Finland (FIMM, Helsinki, Finland) in June 2014. The cells were tested negative for mycoplasma using an IDEXX PCR test and murine pathogens by IDEXX Laboratories Inc (Ludwigsburg, Germany). The viability of cells was tested before and after the inoculations.

Intratibial LNCaP model

Male non-obese diabetic (NOD) mice (25 g at the beginning of the study) with severe combined immunodeficiency (scid) (NOD.scid; NOD.CB17/*Prkdc*$^{scid/scid}$/Rj, Janvier Laboratories, France) were earmarked, housed in individually ventilated cages (IVC), fed an irradiated soy free diet (Teklad Global Diets 2916, Envigo, Madison, WI, USA) and autoclaved tap water *ad libitum*. The minimum quarantine and acclimatization period for the mice was 5 days. For intratibial inoculations and imaging, the mice were anesthetized with inhalation of isoflurane (IsoFlo vet 100%, Zoetis Finland Oy, Helsinki, Finland). Analgesia was provided with buprenorphine (Temgesic 0.3 mg/mL, Indivior Europe Ltd, Dublin, Ireland): 0.1 mg/kg, subcutaneously (s.c.) before and 0.02 mg/mL in drinking water for 2 days after the intratibial inoculation. All inoculations were done using two separate cell batches in one day and the sufficient viability of the cells was confirmed before inoculation. The animals were weighed twice a week and observed daily to monitor the progression of disease. Appearance of any clinical signs were recorded on follow-up forms. First PSA measurements were performed six weeks after the inoculation. At the end of the study, the mice were sacrificed by CO_2 followed by cervical dislocation. At necropsy, all macroscopic findings were recorded. The bones were collected into separate liquid scintillation vials with 10% neutral buffered formalin (NBF), and changed to 70% ethanol on the following day.

Bone labeling and histomorphometry

For measuring dynamic bone histomorphometry parameters, bone was labeled by an injection of fluorescence dye twice during the treatment period of the study. Solid calcein green (#C0875; Sigma-Aldrich, St. Louis, MO, USA) and solid alizarin red (#A3882; Sigma-Aldrich) and were dissolved in 0.9% NaCl and 2% sodium bicarbonate, respectively. Subsequently, the dosing solutions containing 2 mg/mL of calcein green and 3 mg/mL of alizarin red were sterile-filtered. Bone labeling was performed with calcein green (10 mg/kg, s.c.) seven days before sacrifice and with alizarin red (30 mg/kg, s.c.) two days before sacrifice.

Static and dynamic bone histomorphometry parameters were analyzed using an OsteoMeasure7 histomorphometry system (OsteoMetrics, Atlanta, GA, USA) in axial skeleton [1, 2]. Bone samples were dehydrated in ethanol, defatted in xylene, and embedded in methyl methacrylate-based plastic. After the embedding, longitudinal

sections were obtained from a standardized site of proximal tibia using a fully motorized rotary microtome and a tungsten-carbide knife. Static trabecular bone parameters were determined in two 4-μm-thick sections stained in Masson-Goldner's trichrome and dynamic trabecular bone parameters in two unstained 8-μm-thick sections. Cross-sectional cylinders were prepared from a standardized site of tibial shaft in a transverse plane using a linear precision saw and a diamond blade. Both static and dynamic cortical bone parameters were determined in an unstained 200-μm-thick cylinder. All parameters were analyzed following the guidelines of the American Society for Bone and Mineral Research (ASBMR) [3], including the reported parameters summarized in **Table S1**.

Histology

Tumor-bearing tibiae were cut from the diaphysis to save bone samples for cortical histomorphometry. The proximal end of the tibia was processed to plastic. Longitudinal 4-μm sections were obtained and stained with Masson–Goldner trichrome like previously described for histomorphometry. The slides were scanned using a Pannoramic 1000 slide scanner (3DHISTECH Ltd, Budapest, Hungary) and analyzed using Caseviewer software (3DHISTECH Ltd).

SUPPLEMENTARY REFERENCES

1. Dempster, D. W., Histomorphometric analysis of bone remodeling. In *Principles of bone biology*, John P. Bilezikian, L. G. R., T. John Martin, Ed. Academic Press, San Diego: San Diego, CA, USA, **2008**; pp 447-463.
2. Erben, R. G.; Glosmann, M., Histomorphometry in rodents. *Methods Mol Biol* **2012,** 816, 279-303.
3. Dempster, D. W.; Compston, J. E.; Drezner, M. K.; Glorieux, F. H.; Kanis, J. A.; Malluche, H.; Meunier, P. J.; Ott, S. M.; Recker, R. R.; Parfitt, A. M., Standardized nomenclature, symbols, and units for bone histomorphometry: a 2012 update of the report of the ASBMR Histomorphometry Nomenclature Committee. *J Bone Miner Res* **2013,** 28, (1), 2-17.

SUPPLEMENTARY TABLES AND FIGURES

Table S1: Dynamic and static bone histomorphometry parameters measured in tumor-bearing tibiae

Method	Parameter	Standard unit
Dynamic	Trabecular bone formation rate per bone surface	BFR/BS ($\mu m^3/\mu m^2/y$)
	Periosteal bone formation rate per bone surface	BFR/BS ($\mu m^3/\mu m^2/y$)
	Endocortical bone formation rate per bone surface	BFR/BS ($\mu m^3/\mu m^2/y$)
	Trabecular mineralizing surface per bone surface	MS/BS (%)
	Periosteal mineralizing surface per bone surface	MS/BS (%)
	Endocortical mineralizing surface per bone surface	MS/BS (%)
	Trabecular mineral apposition rate	MAR (μm/d)
	Periosteal mineral apposition rate	MAR (μm/d)
	Endocortical mineral apposition rate	MAR (μm/d)
Static	Trabecular bone volume	BV/TV (%)
	Trabecular thickness	Tb.Th (μm)
	Osteoblast number in ratio to tissue area	N.Ob/T.Ar (mm^{-1})
	Osteoclast number in ratio to bone perimeter	N.Oc/T.Ar (mm^{-1})

BFR, bone formation rate; BS, bone surface; BV, bone volume; MAR, mineral apposition rate; MS, mineralizing surface; N.Ob, osteoblast number; N.Oc, osteoclast number; T.Ar, tissue area; Tb.Th, trabecular thickness; TV, tissue volume

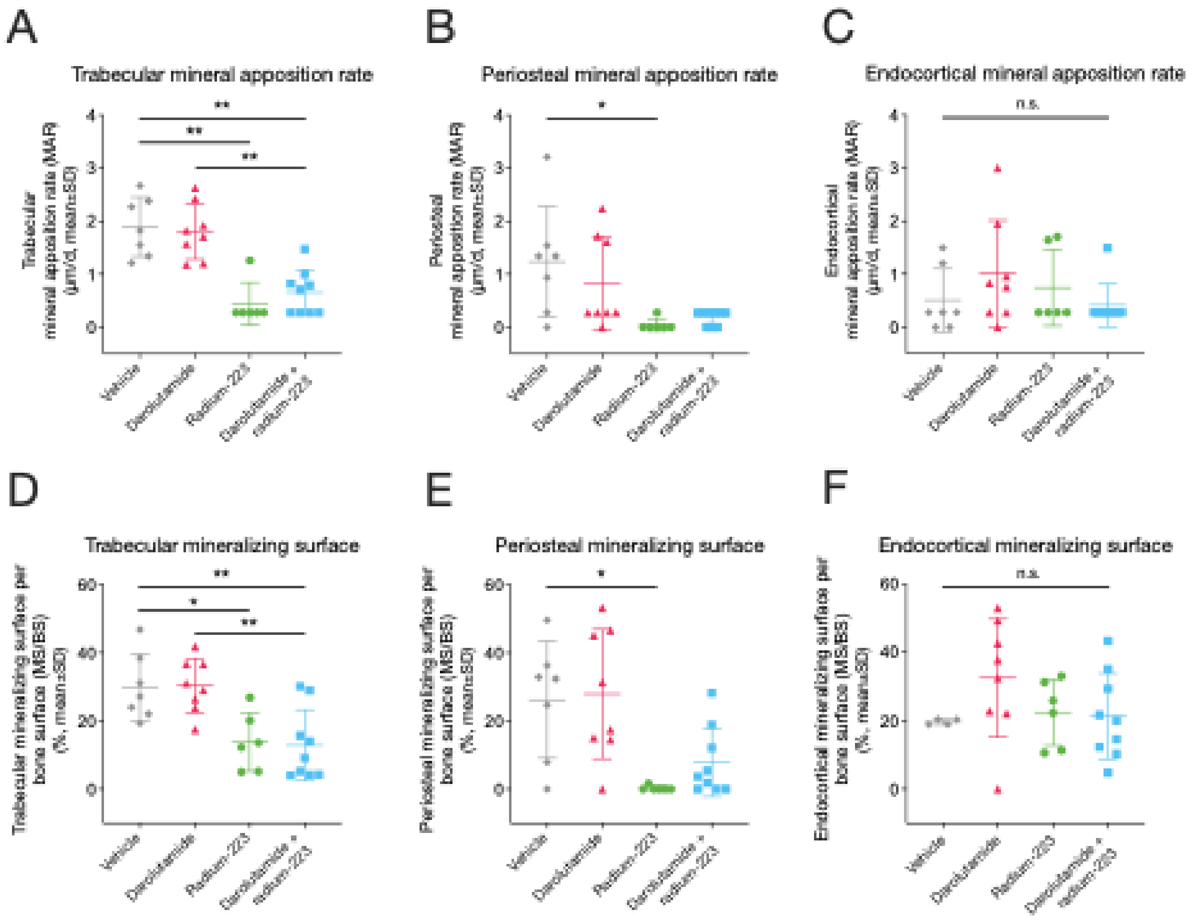

Figure S1: Dynamic bone histomorphometry parameters in tumor-bearing tibiae. (A) Trabecular, (B) periosteal and (C) endocortical mineral apposition rate, and (D) trabecular, (E) periosteal and (F) endocortical mineralizing surface per trabecular, periosteal and endocortical bone surface (MS/BS), respectively, were measured in tumor-bearing tibiae by dynamic histomorphometry. Values describe mean and standard deviation (SD). Statistical analyses were performed using a Kruskal–Wallis test followed by Dunn's pairwise comparison test (A–D, F) or ANOVA followed by contrasts (E): *, $p<0.05$; **, $p<0.01$; n.s., non-significant.
